# *Bmp6* Expression in Murine Liver Non Parenchymal Cells: A Mechanism to Control their High Iron Exporter Activity and Protect Hepatocytes from Iron Overload?

**DOI:** 10.1371/journal.pone.0122696

**Published:** 2015-04-10

**Authors:** Marco Rausa, Alessia Pagani, Antonella Nai, Alessandro Campanella, Maria Enrica Gilberti, Pietro Apostoli, Clara Camaschella, Laura Silvestri

**Affiliations:** 1 Division of Genetics and Cell Biology, IRCCS San Raffaele Scientific Institute, Milan, Italy; 2 Vita Salute University, Milan, Italy; 3 Unit of Occupational Health and Industrial Hygiene, Department of Medical and Surgical Specialties, Radiological Sciences and Public Health, University of Brescia, Brescia, Italy; Lady Davis Institute for Medical Research/McGill University, CANADA

## Abstract

*Bmp6* is the main activator of hepcidin, the liver hormone that negatively regulates plasma iron influx by degrading the sole iron exporter ferroportin in enterocytes and macrophages. *Bmp6* expression is modulated by iron but the molecular mechanisms are unknown. Although hepcidin is expressed almost exclusively by hepatocytes (HCs), *Bmp6* is produced also by non-parenchymal cells (NPCs), mainly sinusoidal endothelial cells (LSECs). To investigate the regulation of *Bmp6* in HCs and NPCs, liver cells were isolated from adult wild type mice whose diet was modified in iron content in acute or chronic manner and in disease models of iron deficiency (*Tmprss6* KO mouse) and overload (*Hjv* KO mouse). With manipulation of dietary iron in wild-type mice, *Bmp6* and *Tfr1* expression in both HCs and NPCs was inversely related, as expected. When hepcidin expression is abnormal in murine models of iron overload (*Hjv* KO mice) and deficiency (*Tmprss6* KO mice), *Bmp6* expression in NPCs was not related to *Tfr1*. Despite the low *Bmp6* in NPCs from *Tmprss6* KO mice, *Tfr1* mRNA was also low. Conversely, despite body iron overload and high expression of *Bmp6* in NPCs from *Hjv* KO mice, *Tfr1* mRNA and protein were increased. However, in the same cells ferritin L was only slightly increased, but the iron content was not, suggesting that *Bmp6* in these cells reflects the high intracellular iron import and export. We propose that NPCs, sensing the iron flux, not only increase hepcidin through *Bmp6* with a paracrine mechanism to control systemic iron homeostasis but, controlling hepcidin, they regulate their own ferroportin, inducing iron retention or release and further modulating *Bmp6* production in an autocrine manner. This mechanism, that contributes to protect HC from iron loading or deficiency, is lost in disease models of hepcidin production.

## Introduction

Hepcidin, the master regulator of iron metabolism, is a liver peptide hormone that negatively regulates dietary iron absorption and iron release from macrophages by binding and degrading of the sole cellular iron exporter ferroportin [1]. Although hepcidin activation is mediated by both circulating and liver iron content, how these two signals govern hepcidin changes is not fully clarified. The characterization of hemojuvelin (HJV), the protein mutated in type 2A hemochromatosis [2], as Bone Morphogenetic Protein (BMP)-coreceptor, functionally linked the BMP-Sons of Mothers Against Decapentaplegic (SMAD) pathway to hepcidin and iron regulation [3]. HJV selectively uses the BMP type II receptor ActRIIA [4], highly expressed in the liver, and the type I receptors ALK2 and ALK3 [5]. In the presence of the ligand, constitutively active type II receptor phosphorylates type I receptor, which phosphorylates SMAD1/5/8 proteins (R-SMADs) that in turn interact with SMAD4. The resulting multiprotein complex translocates to the nucleus to activate target genes [6]. Liver conditional inactivation of *Smad4* [7] or *Alk3* [5] causes severe iron overload due to downregulation of hepcidin, similar to the phenotype of *Hjv* ^-/-^ [8,9] and *Hamp* ^-/-^ [10] mice, whereas liver specific deletion of *Alk2* blunts the response of hepcidin to increased iron levels [5].

BMPs are members of the transforming growth factor beta (TGF-beta) superfamily [11]. *In vitro* several BMPs as BMP2, BMP4 and BMP6 activate hepcidin in the presence of HJV [12]. *In vivo*, Bmp6 is the sole BMP that regulates hepcidin expression in mice. *Bmp6* inactivation causes severe iron overload due to strong hepcidin downregulation and ferroportin stabilization, a phenotype comparable to that of *Hjv* ^-/-^ mice [9], suggesting that Bmp2 and Bmp4 do not compensate for the lack of Bmp6. Transcription of *Bmp6* is suppressed in iron deficiency and upregulated in iron overload [13]; this regulation is liver specific [14] and no other tissue modulates *Bmp6* in response to iron, in agreement with the central role of the liver in iron homeostasis.

The liver is composed by several cell types: parenchymal cells, essentially hepatocytes (HCs), and non-parenchymal cells (NPCs). Among the latter Kupffer cells are resident macrophages (constituting 80–90% of body tissue macrophages), sinusoidal endothelial cells (LSECs) have filter functions between blood and hepatocytes and high endocytic capacity for many ligands, hepatic stellate cells (HSc), or Ito cells, localize between the sinusoids and HCs, and are involved in liver fibrosis when activated [15]. Recently, NPCs (KCs, LSECs and HSc) were reported to express high levels of *Bmp6* compared to HCs [16,17], suggesting that they play a role in hepcidin regulation. Here we extend this observation analyzing *Bmp6* expression in isolated liver cell populations of wild type mice after changing the iron status by acute and chronic diets, and in disease models with opposite and pathological hepcidin levels: the iron loaded *Hjv* ^-/-^ mice which have low hepcidin, and the iron deficient *Tmprss6* ^-/-^ mice characterized by high hepcidin levels. We demonstrate that *Bmp6* is highly expressed in NPCs and that chronic changes in iron status induced by diet modulate *Bmp6* in all cell types according to their intracellular iron content that is influenced by the hepcidin effect on their iron export capacity. However, in NPCs cells, characterized by high ferroportin activity, *Bmp6* expression is independent on cell iron content and more related to their iron uptake and release. We also show that *Bmp6* expression in LSECs is independent from HCs iron. In addition, in our *in vivo* models *Bmp6* increase both in parenchymal and NPCs does not induce *Tmprss6* transcription in HCs.

## Experimental Procedures

### Animal and diets

Wild type C57BL/6 male mice, obtained from Charles River, *Tmprss6* KO mice on a mixed 129/Ola X C57BL/6 background [18], and *Hjv* KO mice on an inbred 129S6/SvEvTac background [9] were housed under a standard 12-hour light/dark cycle with water and chow ad libitum in a pathogen-free animal facility of the San Raffaele Scientific Institute in accordance with the European Union guidelines. The study was approved by the Institutional Animal Care and Use Committee of the San Raffaele Scientific Institute (IACUC number: 514). Only male mice were analyzed when 7–8 week old. To induce stable changes of the iron status, 4 week-old C57BL/6 male mice were fed an iron-balanced (IB; carbonile iron 200 mg/kg; SAFE), an iron-loaded (IL; carbonile iron 8.3 g/kg; SAFE), or an iron-deficient diet (ID), with virtually no iron (< 3 mg iron/kg; SAFE) for 3 weeks. To induce acute iron changes, C57BL/6 animals pretreated by 2 weeks ID diet to induce iron depletion, were administered 1 day an IB, ID or IL diet [19]. The animals were anesthetized by isoflurane and were sacrificed by cervical dislocation. All efforts were made to minimize suffering.3 Livers and spleens were snap-frozen for isolation of RNA or dried for tissue iron content analysis. Liver cell populations were separated as described below.

### Analysis of hematological and iron parameters

Hemoglobin (Hb) levels were determined using a Sysmex KX-21 automated blood cell analyser (Sysmex America) from 0.2 mL of blood extracted by caudal puncture from anesthetized mice. Iron parameters were analyzed as previously described [20]. Briefly, transferrin saturation was calculated as the ratio of serum iron and total iron binding capacity levels, using The Total Iron Binding Capacity Kit (Randox Laboratories Ltd.), according to the manufacturer’s instructions.

To measure liver (LIC) and spleen iron content (SIC) tissue samples were dried at 110°C overnight, weighed, and digested in 1 mL of 3M HCl, 0.6M trichloroacetic acid for 20 hours at 65°C. The cleared acid extract was added to 1 mL of working chromogen reagent (1 volume of 0.1% bathophenanthroline sulfate and 1% thioglycolic acid solution, 5 volumes of water, and 5 volumes of saturated sodium acetate). The solutions were then incubated for 30 minutes at room temperature until color development and the absorbance was measured at 535 nm. A standard curve was plotted using the acid solution containing increasing amounts of iron diluted from a stock solution of Titrisol iron standard (Merck, Darmstadt, Germany) [20].

### Liver cells separation

Liver cells were isolated according to Liu et al. [21] with some modifications. Briefly, mice were anesthetized and sacrificed by cervical dislocation. All efforts were made to minimize suffering.3 Livers were perfused in situ through the inferior vena cava with 0.16 mg/mL collagenase IV (Sigma-Aldrich) in an isosmotic saline solution after transection of the portal vein. After perfusion, livers were removed, teased with scalpels and incubated for 10 min at 37°C in a shaking water bath in a collagenase-solution containing DNase I. Cell suspensions were filtered through a 100-μm and a 70-μm cell strainer. Single cell suspension was then centrifuge at 50g for 3 min to pellet HCs. Cell pellet was washed three times with PBS containing 0.1% BSA and supernatants (containing NPCs) were collected. HCs were further purified by centrifugation on 10% Optiprep (1.06 g/mL) at 50 g for 10 min. NPCs were pelleted at 400 g for 10 min, resuspended in Optiprep 17.6% and stratified onto a 8.2% Optiprep. The cell fraction between the interface of the 8.2 and 17.6% Optiprep was enriched in KCs and LSECs. Middle layers were collected, cells were separated by centrifugation, and LSECs were isolated using MACS CD146 MicroBeads (Miltenyi Biotec). KCs were recovered from the flowthrough. Cell purity was validated by mRNA expression of specific genes: *Tmprss6* for HCs, *Cd146* for LSECs, and *Cd45* for KCs.

### Spleen macrophages separation

Mice were anesthetized and sacrificed by cervical dislocation. All efforts were made to minimize suffering.3 Spleen was isolated and spleen capsule was punctured to send the spleen content out. Cells were resuspended in HBSS medium (Gibco Cell Colture, Portland, OR) and centrifuge at 370g for 10 min. Cell pellet was resuspended in HBSS and incubated on ice with 8 volumes of NH_4_Cl for 10 min to lyse erythrocyte. After centrifugation at 370g for 10 min cell pellet was resuspended in HBSS, stratified onto FBS (Gibco Cell Colture, Portland, OR) and centrifuge at 370g for 10 min. Cell pellet was washed two times with PBS containing 0.5% BSA and 2mM EDTA. To isolate spleen macrophage population, cells were incubated with MACS anti-F4/80 biotin conjugated and pulled down with Streptavidin MicroBeads (Miltenyi Biotec). F4/80 negative cells were further incubated with MACS CD11 MicroBeads (Miltenyi Biotec).

### qRT-PCR

RNAs from isolated HCs were extracted using the guanidinium thiocyanate–phenol–chloroform method (Trizol Reagent, Invitrogen), following the manufacturer’s (Invitrogen) recommendations. RNAs from isolated KCs, LSECs and spleen-derived cells were extracted using the RNeasy mini kit (Qiagen), following manufacturer’s instructions. Total RNA (200 ng for KCs and LSECs and 2 μg for HCs) was retro-transcribed to cDNA using the High Capacity cDNA Reverse Transcription Kit (Applied Biosystem), according to the manufacturer's instructions. Gene expression levels were measured by quantitative real-time PCR using TaqMan Gene Expression Master Mix (Applied Biosystem). Primers used for qRT-PCR are listed in the **S1 Table**.

### Western Blot analysis on liver cells

Liver cells were lysed in lysis buffer (200mM Tris-HCl [pH 8]; 1mM EDTA; 100mM NaCl; 10% glycerol; 0.5% NP-40) and proteins extracts were quantified using the Bio-Rad Protein Assay (Bio-Rad) according to the manufacturer’s instructions. Fifty g of protein extracts were re-suspended in Laemmli buffer, incubated 5 minutes at 95°C, loaded onto a 12% SDS-PAGE and then transferred to Hybond C membrane (Amersham Bioscience Europe GmbH) by standard Western blot technique. Membrane were stained with Ponceau staining for protein quantification and then blocked with 2% nonfat milk in TBS (0.5M Tris-HCl [pH 7.4] and 0.15M NaCl) containing 0.1% Tween 20 (TBST). Blocked membranes were then incubated overnight with anti-Tfr1 (1:2000; Zymed Laboratories. Inc., San Francisco) or with anti-FtL (1:1000; Sigma-Aldrich). After washing with TBST, blots were incubated 1 hour with relevant HRP-conjugated antisera in TBST with 2% nonfat milk and developed using a chemoluminescence detection kit (ECL; Amersham Biosciences).

### Electro mobility shift assay (EMSA)

EMSA was performed as already described [22]. Briefly, the ^32^P-labeled IRE probe was generated by *in vitro* transcription of the plasmid pSPT-Fer. Cell extracts (2 μg of total proteins) were incubated with a molar excess of ^32^P-labeled IRE probe (100.000 cpm) in the presence or absence of 2% β-mercaptoethanol. Proteins were separated by non-denaturing polyacrylamide gel electrophoresis and dried gels were exposed to autoradiography. Band intensity of the exposed films was quantified by densitometry.

### Quantification of cellular iron

The concentration of Iron in the different biological matrices examined was measured by inductively coupled plasma mass spectrometry (ICP-MS) using a Perkin Elmer ELAN DRC II instrument (Perkin Elmer Sciex, Woodbridge, ON, Canada). The total quant technique analytical method, with external calibration using a dynamic reaction cell, was adopted. The coefficients of variation ranged from 4.5% to 7.6% among analytical series and from 5% to 10.5% between the series. The instrument was calibrated using standard solution at a concentration of 10 μg/L (Multielement ICP-MS Calibration Standard 3, Matrix per Volume: 5% HNO_3_ per 100 mL, Perkin Elmer Plus).

Each sample underwent two-fold determination. The accuracy of the method was calculated in ultrapure water. Bovine liver standard reference materials (NIST 1640 and MS1577b, respectively, National Institute of Standard and Technology, Gaithersburg, MD, USA) were used to better approximate the results from biological matrices. It ranged between 86.5 and 88.5%. The detection limit, determined on the basis of three standard deviations of the background signal, was determined at 0.005 μg.

### Statistical analysis

Data are presented as mean ± SE. Student’s t-test was used to calculate significance (P<0.05).

## Results

### *Bmp6* is expressed mainly in NPCs and correlates with intracellular iron content

**Fig 1:**
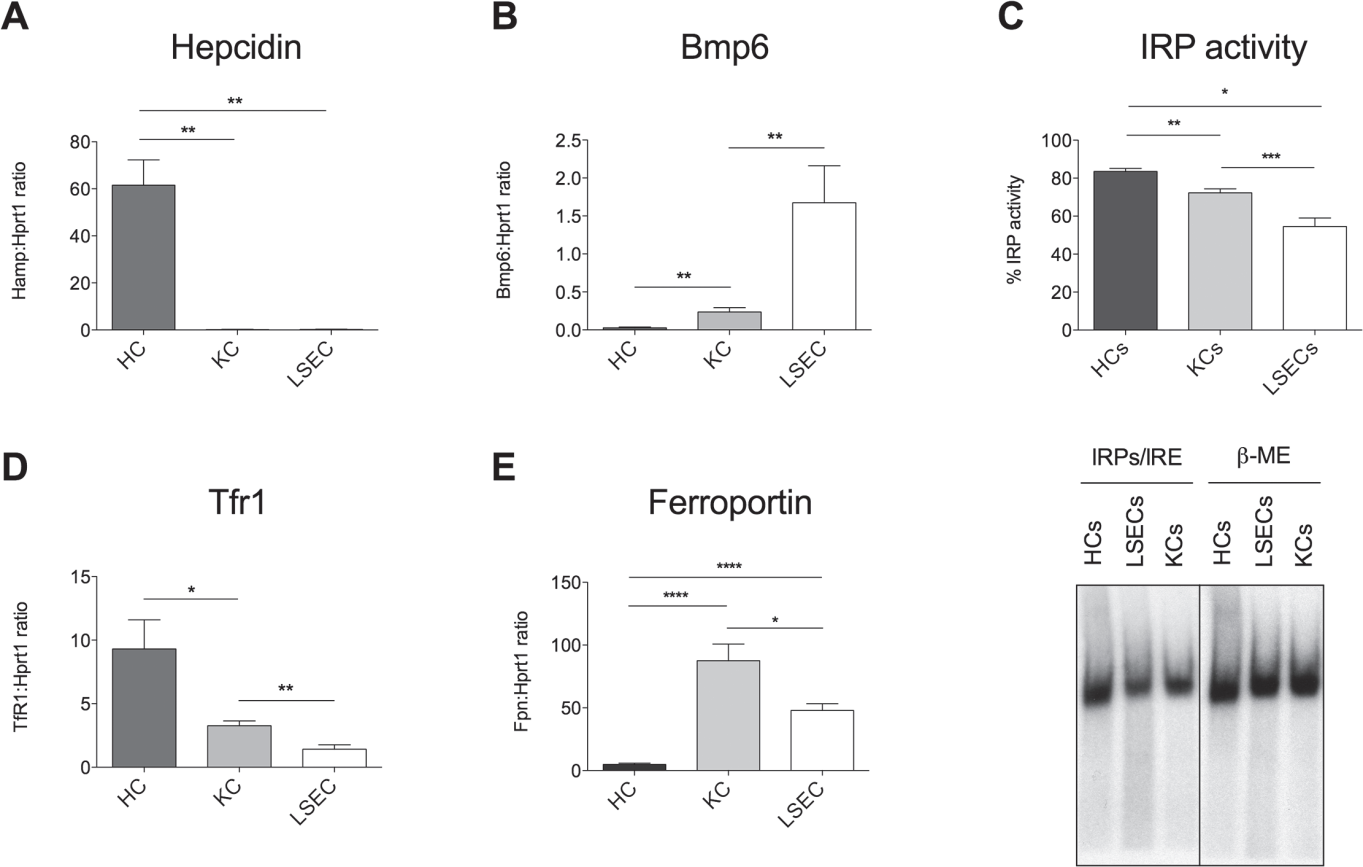
Isolated liver cells characterization. Liver cells were isolated from 4–6 mice using the protocol reported in Experimental Procedures. Hepcidin (*Hamp*, **A**), Bone Morphogenetic Protein 6 (*Bmp6*, **B**), Transferrin Receptor 1 (*Tfr1*, **D**) and ferroportin (*Fpn*, **E**) mRNA expression was quantified by qRT-PCR relative to housekeeping *Hprt1* gene. (**C**) **Upper panel**: IRE/IRP-binding activity of HCs, KCs and LSECs, expressed as percentage of total activity. The plot refers to three independent experiments. **Lower panel**: IRE-IRPs electro mobility shift assay (EMSA). β-ME (beta-mercaptoethanol) was used to evaluate the total binding activity. A representative experiment was shown. Error bars indicate SE. *: P<. 05; **: P<. 01; ***: P<. 001.

To investigate *Bmp6* expression under basal conditions, HCs, KCs and liver sinusoidal LSECs were isolated from adult male mice as described in Material and Methods. HSCs were not included in the analysis because of the low amount of cells we were able to purify from a single animal. The purity of the preparations was assessed by specific markers: *Tmprss6* for HCs, *Cd45* for KCs and *Cd146* for LSECs. HCs and LSECs expressed high levels of *Tmprss6* (**S1A Fig**) and *Cd146* (**S1B Fig**), respectively, whereas *Cd45* (**S1C Fig**) was predominantly expressed in KCs. *Hamp* was exclusively expressed in HCs (**Fig 1A**), whereas *Bmp6* was highly expressed in NPCs, mainly in LSECs (**Fig 1B**). To investigate whether *Bmp6* expression depends on cell iron content, we measured the Iron Regulatory Proteins (IRPs)/Iron Responsive Elements (IRE) binding activity that directly correlates to cell iron [23]. Alternatively we quantified *Tfr1* mRNA that is stabilized by IRPs in iron deficiency and degraded in iron overload and thus indirectly estimates intracellular iron content [23]. In all cell types, *Bmp6* levels inversely correlated with both IRPs binding activity (**Fig 1C**) and *Tfr1* expression (**Fig 1D**). Since *Tfr1* changes are easier to assess and more pronounced than changes of IRPs activity, we used *Tfr1* expression to investigate cells iron content in subsequent experiments. Notably, ferroportin (*Fpn*) expression was low in HCs and high in NPCs, especially in KCs (**Fig 1E**). The latter finding, considering the lower levels of IRPs binding activity in NPCs as compared to HCs (**Fig 1C)**, indicates that NPCs have a prevalent iron exporter functions.

### Chronic dietary iron changes modulate *Bmp6* in all cell types

**Fig 2:**
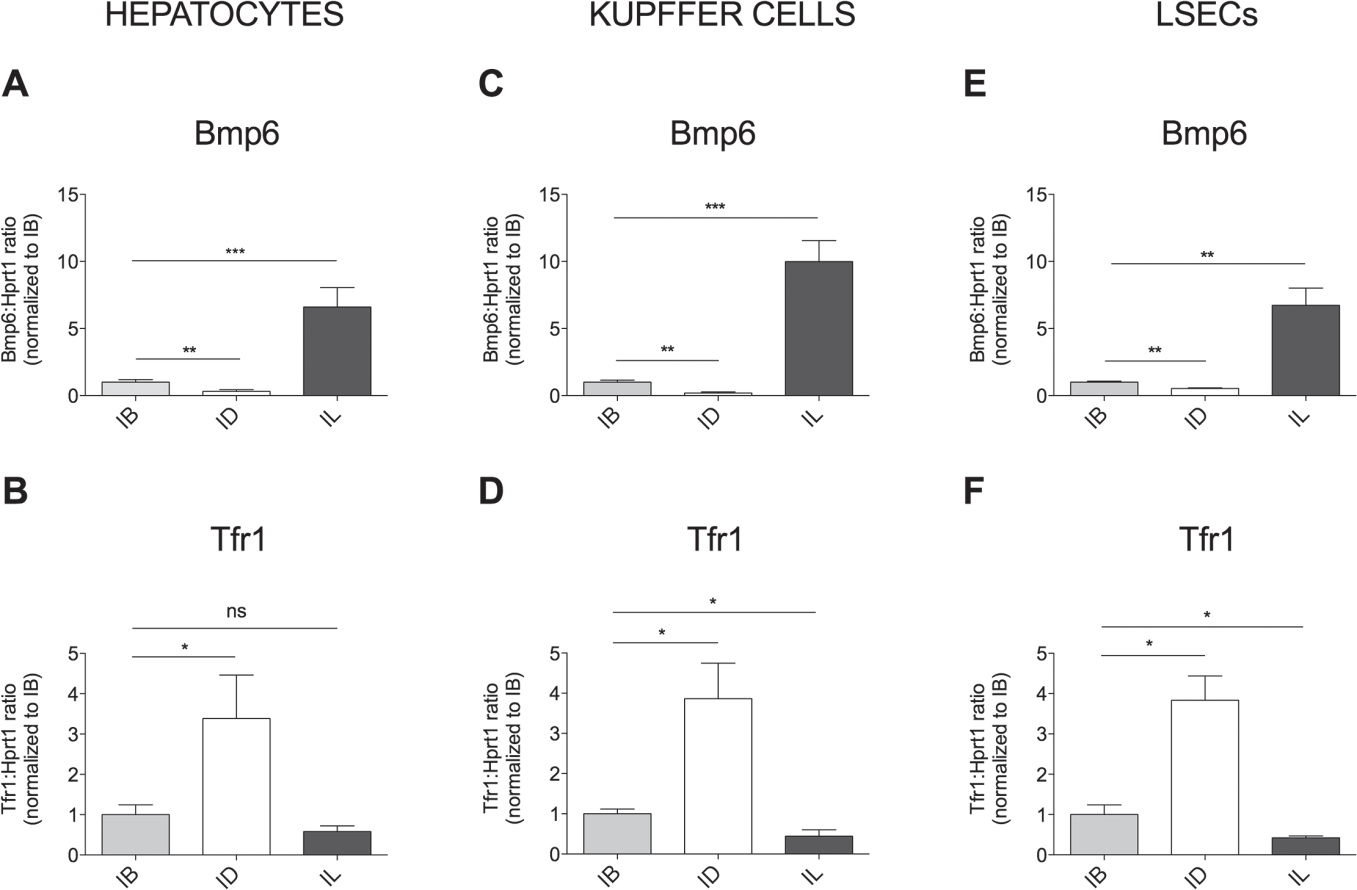
*Bmp6* and *Tfr1* expression in chronic dietary iron changes. *Bmp6* (**A, C, E**) and *Tfr1* (**B, D, F**) mRNA expression was evaluated by qRT-PCR in isolated HCs (**A, B**), KCs (**C, D**) and LSECs (**E, F**) from 4–12 mice/group. mRNA expression ratio was normGalized relative to housekeeping *Hprt1*. Mean control value of IB-treated mice was set to 1. Error bars indicate SE. *: P<. 05; **: P<. 01; ***: P<. 001; ns: not significant.

Liver and spleen are iron storage organs able to accumulate or release iron according to body needs. To investigate whether and how dietary iron changes modulate *Bmp6* expression in liver cells and in spleen macrophages, different cohorts of mice were fed an iron balanced (IB), iron deficient (ID) or iron loaded (IL) diet for 3 weeks. Iron parameters changed as expected: LIC (**S2A Fig**), SIC (**S2B Fig**) and transferrin saturation (TS, **S2C Fig**) were significantly increased and decreased in IL- and ID-treated mice, respectively. Also Hb was significantly increased in animals maintained an IL diet and decreased following an ID diet (**S2D Fig**). In HCs, *Hamp* (**S2E Fig**), Inhibitor of differentiation 1 [*Id1*, a target of the Bmp-Smad pathway] (**S2F Fig**) and *Bmp6* (**Fig 2A** and **S8 Fig**) were transcriptionally modulated in the same direction of iron changes. Both KCs (**Fig 2C**) and LSECs (**Fig 2E**) upregulated *Bmp6* expression in dietary iron overload, while the opposite occurred in iron deficiency. In all cells (Fig 2B, 2D and 2F) *Tfr1* was stabilized in iron deficiency and downregulated in iron overload. We concluded that in wild type animals *Bmp6* is regulated in all liver cell types, according to their intracellular iron content, which reflects the difference between iron uptake and release. A similar regulation does not occur in the spleen: total spleen (**S3A Fig**), spleen-derived macrophages, as F4/80^+^ (**S3B Fig**), Cd11b^+^ (**S3C Fig**) and F4/80^-^ Cd11b^-^ cells (**S3D Fig**), did not change *Bmp6* expression following systemic iron variations.

### Changes in hepcidin expression do not require a change in LSECs *Bmp6* expression in response to acute mild iron changes

**Fig 3:**
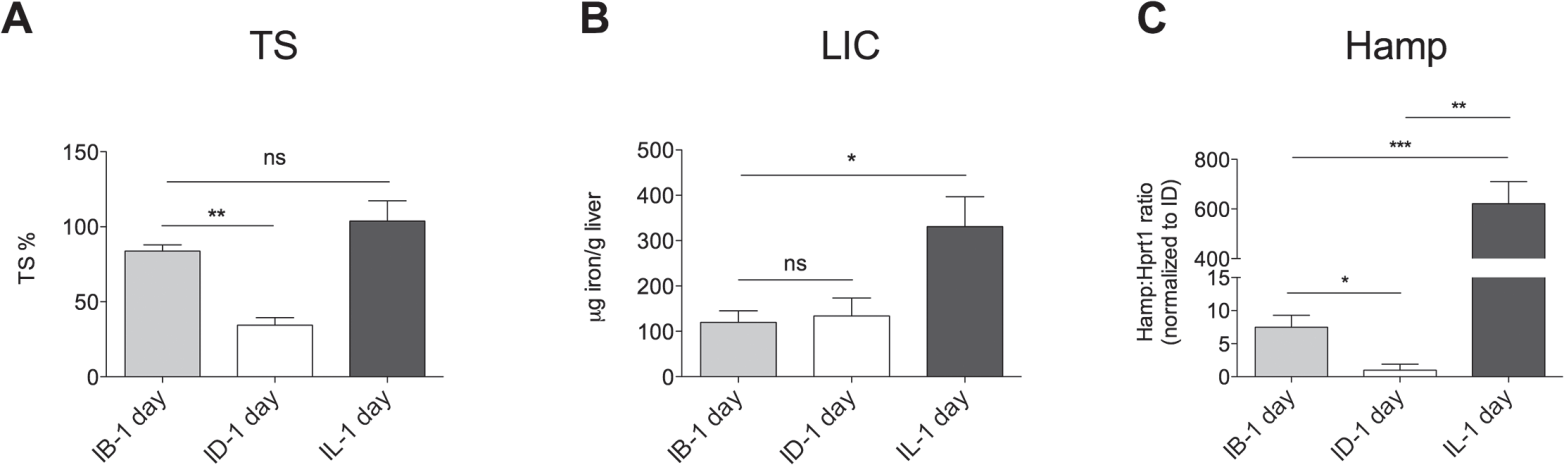
Iron parameters in acute dietary iron changes. Transferrin saturation (TS, **A**) and non-heme liver (LIC, **B**) iron content were measured in mice (6–8/group) treated by an iron deficient diet for two weeks and then challenged with 1 day ID (ID-1day), iron balanced (IB-1day) and iron loaded (IL-1 day) diets. *Hamp* (**C**) expression was evaluated in HCs isolated from 4–7 mice/group. *Hprt1* was used as housekeeping gene. mRNA expression ratio was normalized to control (ID-1 day) mean value set to 1. Error bars indicate SE. *: P<. 05; **: P<. 01; ***: P<. 001; ns: not significant.

**Fig 4:**
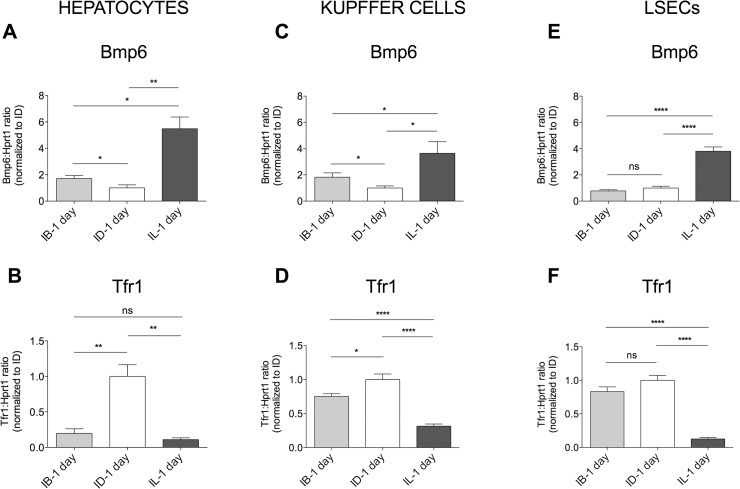
*Bmp6* and *Tfr1* expression levels in acute dietary iron changes. *Bmp6* (**A, C, E**) and *Tfr1* (**B, D, F**) mRNA expression was evaluated by qRT-PCR in HCs (**A, B**), KCs (**C, D**) and LSECs (**E, F)** isolated from 4–7 mice/group. *Hprt1* was used as housekeeping gene. mRNA expression ratio was normalized to control (ID-1 day) mean value set to 1. Error bars indicate SE. *: P<. 05; **: P<. 01; ***: P<. 001; ns: not significant.

To investigate the kinetic of *Bmp6* response after acute dietary iron variation, mice were iron-depleted by a low-iron diet for 2 weeks and maintained for 1 day the same diet (< 3 mg/kg iron, defined as ID in **Figs 3 and 4**), or switched to a standard diet (200 mg/kg iron, defined as IB in **Figs 3 and 4**) or to a rich iron diet (8.3 mg/kg iron, defined as IL in **Figs 3 and 4**).

1 day ID, standard (IB) or IL diet, according to described protocols [19]. Results after IL diet were as expected: TS (**Fig 3A**), LIC (**Fig 3B**) and *Hamp* (**Fig 3C**) were all increased, *Bmp6* was increased and *Tfr1 mRNA* decreased in all cell types (**Fig 4**). In animals switched to a standard (IB) diet, the response of circulating and intracellular iron was uncoupled: TS was increased (**Fig 3A**), whereas LIC (**Fig 3B**) was unchanged and *Hamp* was only moderately increased compared with animals maintained an ID diet (**Fig 3C**). HCs and KCs *Bmp6* was concordant with *Hamp* and moderately increased in comparison with ID (Fig 4A and 4C). Remarkably, LSECs *Bmp6* remained low as in ID (**Fig 4E**).

Considering intracellular iron we observed a discrepancy between LIC and *Tfr1* mRNA: although LIC was similar in IB and ID (**Fig 3B**), HCs *Tfr1* was strongly induced only in ID (**Fig 4B**) and downregulated in IB as well as in IL mice, which had the highest LIC (**Fig 3B**). This observation suggests that a threshold of intracellular iron should likely be achieved to induce reduction of *Tfr1* expression and that *Tfr1* mRNA is a better sensor of iron decrease than increase.

KCs behaved as HCs: in IB *Bmp6* was slightly upregulated (**Fig 4C**) and *Tfr1* mRNA mildly decreased (**Fig 4D**) compared to ID mice. However, in LSECs both *Bmp6* (**Fig 4E**) and *Tfr1* expression (**Fig 4F**) were comparable between ID and IB mice, suggesting that, although iron absorption increases significantly when animals previously iron depleted are switched to 1 day IB diet [19], this is not sufficient to activate *Bmp6* in LSECs, the activation requiring higher iron burden, as occurs in IL diet (**Fig 4E**).

### *Bmp6* regulation in the iron loaded *Hjv* KO mice

To investigate the regulation of *Bmp6* in pathological iron overload, we analyzed *Hjv* KO mice, characterized by severe iron overload with increased TS (**S4A Fig**) and LIC (**S4B Fig** and [8,9]), due to inactivation of the Bmp6-coreceptor *Hjv* that results in strong *Hamp* reduction (**S4C Fig**). Severe hepcidin insufficiency causes ferroportin stabilization and, although iron is increased in the circulation and in the liver, the spleen is iron poor (**S4D Fig**) [8,9].

**Fig 5:**
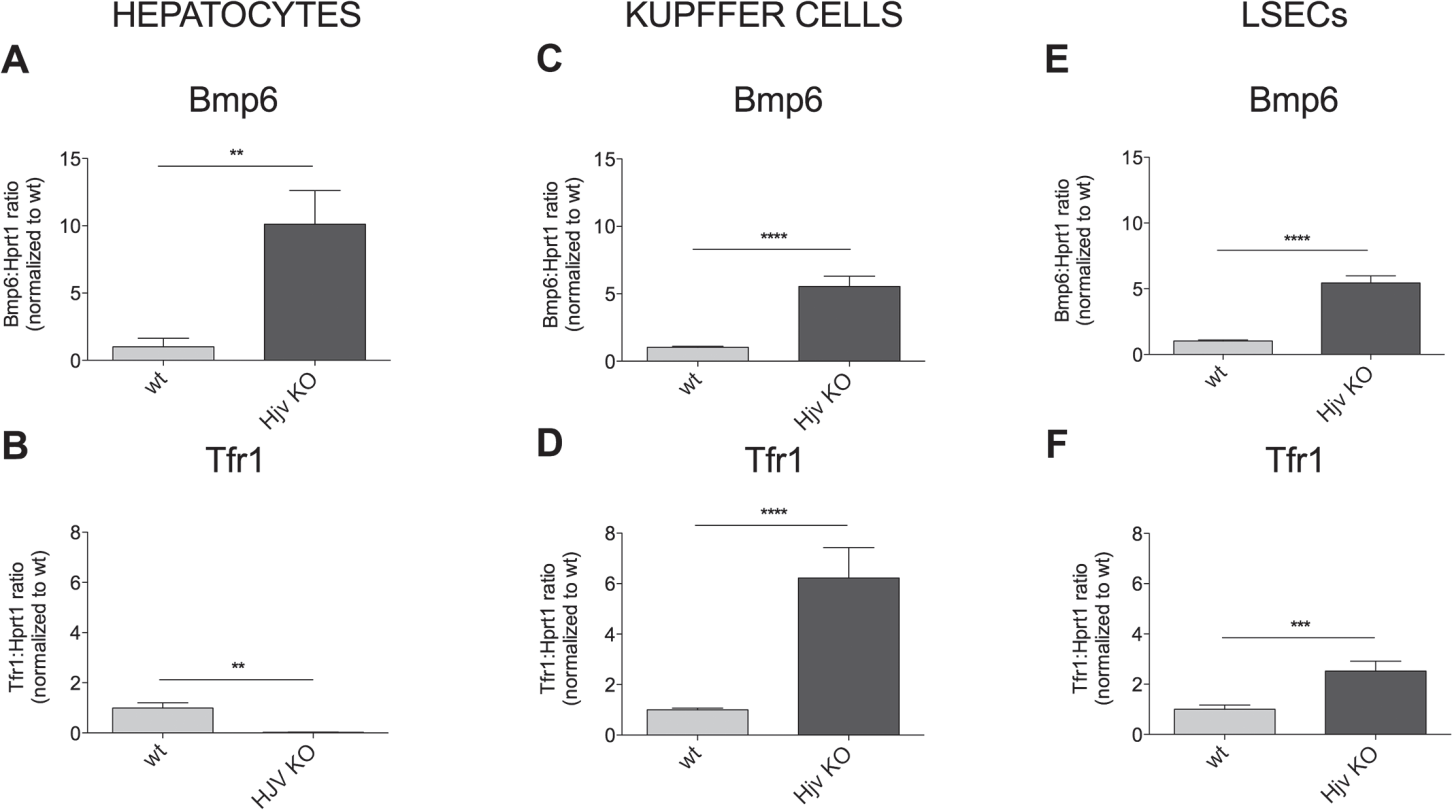
*Bmp6* and *Tfr1* expression in *Hjv* KO mice. *Bmp6* (**A, C, E**) and *Tfr1* (**B, D, F**) mRNA expression was evaluated in liver cells isolated from 6–10 mice by qRT-PCR, using *Hprt1* as the referred housekeeping gene. mRNA expression ratio was normalized to control wild type (wt) mean value set to 1. Error bars indicate SE. **: P<. 01; ***: P<. 001; ****: P<. 0001.

In *Hjv* KO HCs *Bmp6* mRNA was increased (**Fig 5A** and **S8 Fig**) and *TfR1* expression was suppressed (**Fig 5B**), compatible with high iron content, as demonstrated also by degradation of Tfr1 protein (**S5A Fig**). *Bmp6* was increased also in KCs and LSECs (Fig 5C and 5E). However, unlike HCs, *TfR1* was up-regulated in NPCs both at mRNA (Fig 5D and 5F) and protein levels (S5B and S5C Fig).

To determine the iron content in *Hjv* KO liver cells populations, HCs, KCs and LSECs were processed by inductively coupled plasma mass spectrometry (ICP-MS). HCs from *Hjv* KO mice are severely iron loaded, as expected (**Table 1**). In contrast, iron levels in KCs and LSECs are comparable to the iron replete wild type littermates (**Table 1**), likely because of their elevated iron export ability due to low hepcidin and ferroportin stabilization. To further investigate the regulation of iron-related proteins in this model, we analyzed ferritin L (FtL) levels by Western Blot analysis. FtL is strongly increased in *Hjv* KO HCs (**S5A Fig**) as expected due to high iron levels. However FtL is increased also in *Hjv* KO KCs and LSECs (S5B and S5C Fig), which had intracellular iron comparable to wild type animals (**Table 1**).

**Table 1:** Total iron quantification in liver cells from wild type and *Hjv* KO mice.

	**HCs**	**KCs**	**LSECs**
**wild type**	2.127±0.442	0.115±0.096	0.248±0.045
***Hjv* KO**	125.639±87.937*	0.405±0.252^ns^	0.481±0.267^ns^

HCs: hepatocytes. KCs: kupffer cells. LSECs: liver sinusoidal endothelial cells.

*: *Hjv* KO vs wild type *P* = 0.036.

^ns^: *Hjv* KO vs wild type non significant.

### Regulation of *Bmp6* in the iron deficient *Tmprss6* KO mice

The *Tmprss6* KO mouse model is characterized by iron deficient anemia, as assessed by low hemoglobin (**data not shown**), low LIC (**S6A Fig**), low TS (**S6B Fig**), due to hyperactivation of the BMP-SMAD pathway [24] and inappropriately high hepcidin levels (**S6C Fig** and [18]). Spleen iron of *Tmprss6* KO mice is comparable with that of wild type littermates (**S6D Fig**), thus inappropriate for its condition of iron deficiency.

**Fig 6:**
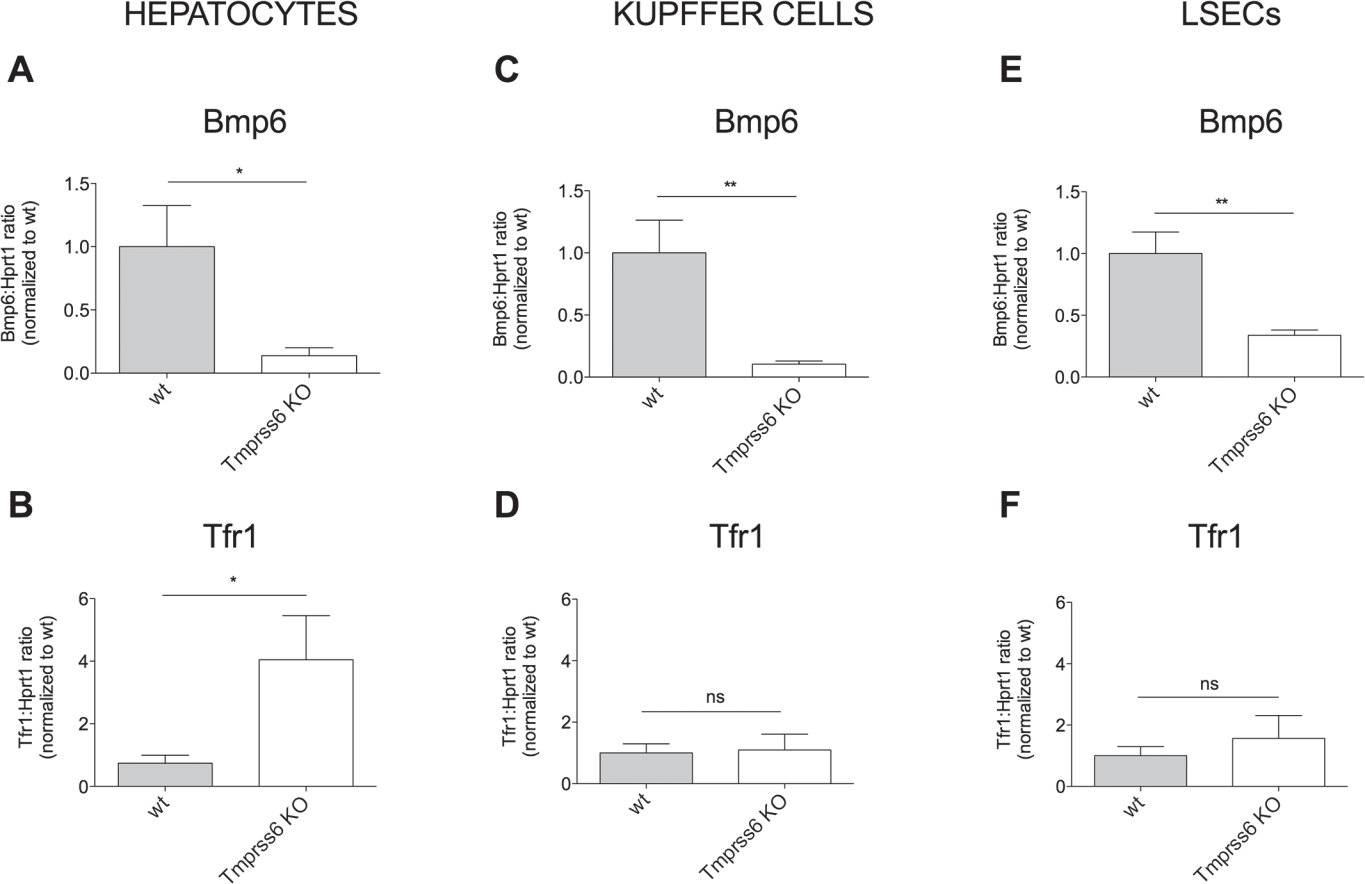
*Bmp6* and *Tfr1* expression in *Tmprss6* KO mice. *Bmp6* (**A, C, E**) and *Tfr1* (**B, D, F**) expression was measured in liver cells isolated from wild type (wt) and *Tmprss6* KO mice (4–6 mice/group) by qRT-PCR, using *Hprt1* as housekeeping gene. mRNA expression ratio was normalized to wt mean value set to 1. Error bars indicate SE. *: P<. 05; **: P<. 01; ns: not significant.

In isolated HCs *Bmp6* expression was low (**Fig 6A)** and *TfR1* elevated (**Fig 6B**). However, *Tfr1* expression in KCs (**Fig 6D**) and LSECs (**Fig 6F**) was similar to wild type littermates, reflecting spleen iron content (**S6D Fig**) and compatible with iron retention due to high hepcidin. *Bmp6* in KCs (**Fig 6C**) and LSECs (**Fig 6E**) was significantly lower than in wild type cells, as in HCs. In analogy with the *Hjv* KO model but in opposite direction, these results suggest that NPCs modulate *Bmp6* in a way independent on their iron content, in this case reflecting low iron uptake.

### *Tmprss6* expression is independent of *Bmp6* expression

Recently *Bmp6* was shown to be a positive regulator of *Tmprss6* expression in mice treated with prolonged iron rich diet [25]. *Tmprss6* up-regulation was interpreted as a negative feedback mechanism to avoid excessive hepcidin production in response to iron increase [25]. We took advantage of the analyzed models to investigate whether *Bmp6* produced by HCs and/or NPCs could play a role in the regulation of *Tmprss6*. As shown in **S7 Fig,** in our models we did not observe a transcriptional activation of *Tmprss6* in all high *Bmp6* conditions, including dietary both chronic (**S7A Fig)** and acute (**S7B Fig**) and disease-associated (**S7C Fig**) iron overload. Only a trend toward a slight increase was observed in chronic IL diet.

## Discussion

The liver hormone hepcidin controls systemic iron homeostasis in response to different stimuli, such as variations of total body iron, erythropoiesis expansion, hypoxia and inflammation. In response to iron increase, hepcidin is up-regulated by the BMP-SMAD signaling pathway, activated by BMP6 and inhibited by the serine protease TMPRSS6 [26]. At the same time iron regulates *BMP6* expression in a homeostatic manner [13]. The orchestration of iron metabolism by Bmp6 is a liver-specific function, since systemic iron changes do not modify *Bmp6* expression in other organs [14]. However, how *Bmp6* expression is upregulated by iron remains elusive and the specific contribution of the different liver cell types in *Bmp6* regulation in physiological and pathological conditions remains poorly understood.

In the present study we investigated the *Bmp6* expression in isolated liver cells, in response to physiological iron changes and in murine models of iron disorders. We also attempted to compare cell iron content and *Bmp6* expression in the different cell types. As a measure of iron content we used *Tfr1* mRNA, since in basal conditions its transcriptional regulation corresponds to the IRP1activity (**Fig 1C**) in the different cell types.

We confirm that *Hamp* expression in basal conditions is high although HCs *Bmp6* is low, compatible with a paracrine hepcidin stimulation by the elevated NPCs *Bmp6*, as previously observed [27]. Interestingly, KCs and LSECs have lower IRP binding activity than HCs and express higher levels of ferroportin, findings compatible with an important function of iron exporters. At variance with published data [27] we observed that *Bmp6* changes are consensual to systemic and intracellular iron in all liver cells, including HCs, when variations of the iron status are stably induced by the chronic (3-weeks) diet. We propose that in steady state the equilibrium is reached between iron uptake and hepcidin-mediated iron export and that *Bmp6* expression reflects this equilibrium in all liver cells, including NPCs. We excluded that in the same chronic conditions spleen macrophages participate to *Bmp6* regulation, confirming the central role of liver *Bmp6* in hepcidin control.

Acute dietary iron changes in iron-depleted mice caused marked variations of *Hamp* and *Bmp6* in all liver cells only when animals were exposed to the iron-enriched (IL) diet. In IB-treated ones TS increased but *Bmp6* increase was mild in HCs and KCs and absent in LSECs, that remain relatively iron free likely because of their rapid iron release. In the interpretation of our results, the dynamics of iron uptake and release should be considered, the latter being more important in NPCs than in HCs, because of their higher ferroportin expression. It should also be noted that in the acute model of iron variation the effect of hepcidin on ferroportin is not as evident as in chronic models.

In the 1-day diet, despite HCs iron and *Bmp6* are both increased in IB-treated mice, LSECs do not up-regulate *Bmp6*. Thus LSECs do not respond to acute and likely modest and transient dietary iron changes.

A signal from HCs that drives *Bmp6* activation in NPCs as been previously proposed [27]. Our results suggest that *Bmp6* expression in LSECs increases only when circulating iron is stably high as in IL diet.

Disease models of iron overload (*Hjv* KO) and deficiency (*Tmprss6* KO) are especially informative to define *Bmp6* regulation. Since characterized by abnormal hepcidin response, opposite to the physiological one, these models dissociate *Bmp6* expression from hepcidin response. *Hjv* KO mice have appropriately high *Bmp6*, as observed [19] but low hepcidin, and *Tmprss6* KO have the opposite phenotype of high hepcidin and low *Bmp6*. In our hands the direct correlation between iron (*Tfr1* mRNA) and *Bmp6* is maintained in HCs, but not in NPCs whose *Tfr1* expression was similar to that of spleen macrophages. Although HCs have high (in case of *Hjv* KO) or low (in case of *Tmprss6* KO) iron content, KCs and LSECs show high *Tfr1* in *Hjv* KO mice, and low *Tfr1* in *Tmprss6* KO animals, while *Bmp6* is upregulated in the former, and downregulated in the latter in all liver cells examined. To clarify the discrepancy observed between *Tfr1* and *Bmp6* we measured Tfr1 and ferritin L protein and intracellular iron content in isolated liver cells from *Hjv* KO mice, which showed the more striking discrepancy between *Tfr1* and *Bmp6*. We found that both Tfr1 and ferritin L proteins were increased in NPCs although the iron content was not significantly different from wild type cells but strikingly lower than HCs iron (**Table 1**). To reconcile these data with the high levels of Tfr1 we reasoned that the iron taken up from the circulation is sequestered into ferritin and rapidly exported by Fpn (not degraded by hepcidin), leaving low the labile iron pool. The effect of abnormal hepcidin levels is quite evident in NPCs also in *Tmprss6* KO mice. Since NPCs opposite to HCs, have high ferroportin expression and considering the iron status of the models studied, is plausible to conclude that *Bmp6* expression in NPCs reflects their iron flux (high in *Hjv* KO and low in *Tmprss6* KO).

It has been suggested that ferritin from HCs may act as a paracrine molecule to regulate *Bmp6* expression in LSECs [28]. Both HCs and NPCs regulate *Bmp6* expression apparently in response to changes of HCs iron, in chronic conditions. This has been proposed to require a signal/cytokine to transmit information on the iron state from HCs to NPCs [27]. We favor a different interpretation based on the following observations. First, a mild HCs iron increase in acute dietary iron treatment does not activate *Bmp6* in LSECs. Second, *Fpn* inactivation in HCs, macrophages and duodenum, that leads to liver iron overload and decreases serum iron and TS, does not increase, but even down-regulate, liver *Bmp6* expression [29]. In this mouse model, KCs are highly iron loaded, whereas HCs show only mild iron accumulation. However, Bmp6 is downregulated in the total liver of these mice. These results agree with our model. In mice with conditional deletion of *Fpn* HCs iron does not regulate *Bmp6* expression that is low despite the mild iron increase. Moreover this mice model is compatible with the concept that iron flux (the uptake by Tfr1 and the export by ferroportin) regulates *Bmp6* expression. With conditional deletion of *Fpn* the uptake of iron is low because of low TS, and the export is blocked because of Fpn inactivation.

We observe that NPCs *Bmp6* is discrepant with iron content measured as *Tfr1* mRNA (or protein) in several conditions. In the interpretation of the results, the higher ferroportin expression of NPCs in comparison with HCs should be considered. Our results are compatible with the possibility that NPCs regulate *Bmp6* expression sensing the iron flux, while *Tfr1* reflects the labile iron pool resulting from the uptake/release balance, the latter being highly dependent on hepcidin itself and more evident when levels of hepcidin are stable in chronic conditions. This would explain why *Bmp6* is low in *Tmprss6* KO (where iron uptake is minimal, but the release is reduced by ferroportin degradation) and high in *Hjv* KO, where the uptake is massive but massive is also the export, due to the hepcidin deficiency. These disease models highlight the physiological crosstalk of liver cells, because of their altered hepcidin response.

**Fig 7:**
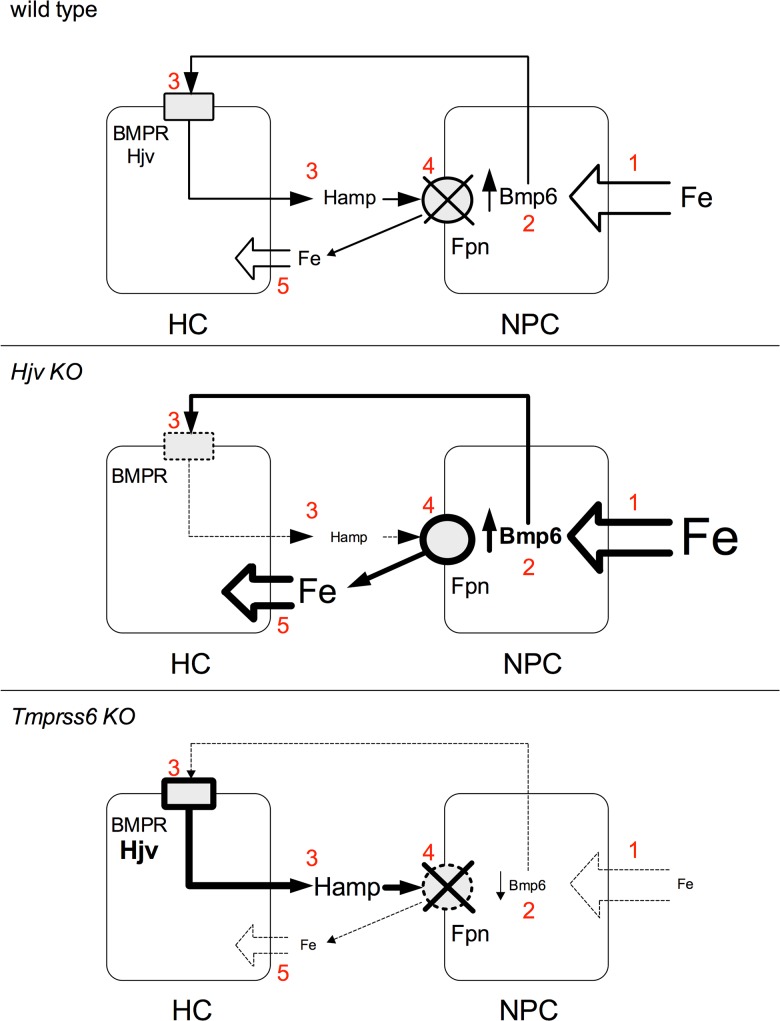
Proposed model of the crosstalk between *Bmp6*-producing NPCs and *hepcidin*-producing HCs in different conditions. **(1)** NPC iron entry; **(2)** Bmp6 production; **(3)** Bmp6-mediated activation of the BMPR-Hjv complex and hepcidin production; (**4**) hepcidin-mediated degradation of ferroportin; **(5)** HC iron entry. The thickness of solid lines and arrows is proportional to the amount of iron, Bmp6, ferroportin and hepcidin. The dotted line indicates inhibition of the pathway. HC: hepatocyte; NPCs: non parenchymal cells (Kupffer cells and sinusoidal endothelial cells); Hamp: hepcidin; BMPR: Bone Morphogenetic Protein Receptor; Hjv: hemojuvelin; Fpn: ferroportin.

In conclusion we have strengthened the crosstalk among the different liver cell types and showed a crucial role of NPCs in the modulation of *Bmp6* both in physiological and pathological conditions. Although HCs are able to increase *Bmp6* in iron overload, the major contribution is provided by the high *Bmp6*-expressing NPCs, especially LSECs, likely because they are in direct contact with the circulation and may behave as a liver iron sensor. Increasing hepcidin through Bmp6, they contribute not only to systemic, but also to local iron regulation, withholding iron, as a possible protective mechanism of HCs (**Fig 7**). In *Hjv* KO model *Bmp6* production persists, but lack of hepcidin response abolishes the protective effect of LSECs and KCs on HCs iron loading. In *Tmprss6* KO mice *Bmp6* expression is low, irrespective of the relatively high iron content of NPCs that is determined by the high hepcidin levels. This iron sequestration contributes to worsen systemic and HCs iron deficiency, as illustrated in **Fig 7**.

We have uncovered a previously unrecognized mechanism of controlled storage and release of iron by NPCs: the positive feedback between *Bmp6* expression in these cells and ferroportin degradation by the induced hepcidin would allow these cells to accumulate iron for later needs and at the same time to protect HCs from iron overload.

## Supporting Information

S1 Fig Isolated liver cells characterization. Liver cells were isolated from 4 mice. *Tmprss6* (**A**), *Cd146* (**B**) and *Cd45* (**C**) mRNA expression was quantified by qRT-PCR relative to housekeeping *Hprt1* mRN*A* to evaluate the purity of HCs, KCs and LSECs, respectively. Error bars indicate SE. *: P<. 05; **: P<. 01; ***: P<. 001.
(TIFF)

S2 Fig Iron parameters and hepatocyte iron-related genes in chronic dietary iron changes. Mice were fed an iron balanced (IB), iron deficient (ID) and iron loading (IL) diet for 3 weeks (6–8 mice/group). Non-heme liver (LIC, **A**), spleen (SIC, **B**) iron content, transferrin saturation (TS, **C**) and hemoglobin levels (Hb, **D**) are shown. In isolated hepatocytes hepcidin (*Hamp*) and *Id1* mRNA expression was quantified by qRT-PCR relative to housekeeping *Hprt1* gene. mRNA expression ratio was normalized setting control (IB) mean value to 1. Error bars indicate SE. *: P<. 05; **: P<. 01; ***: P<. 001.
(TIFF)

S3 Fig *Bmp6* expression in total spleen and spleen-derived cells. Spleen and spleen-derived cells were isolated from mice maintained an iron balanced (IB), iron deficient (ID) and iron loading (IL) diet for 3 weeks. *Bmp6* expression from total spleen (**A**, 3–4 mice), from F4/80^+^ cells (**B**, 6 mice), from Cd11b^+^ cells (**C**, 6 mice) and from negative fractions (**D**, 6 mice) was quantified by qRT-PCR relative to *Hprt1* as the housekeeping gene. Error bars indicate SE. ns: not significant.
(TIFF)

S4 Fig Iron parameters and hepcidin levels in *Hjv* KO mice. Transferrin saturation (TS, **A**), non-heme sliver iron content (LIC, **B**) and non-heme spleen iron content (SIC, **D**) were measured in wild type (wt) and *Hjv* KO animals (4 mice/group). In isolated HCs, hepcidin (*Hamp*, **C**) expression was measured by qRT-PCR, using *Hprt1* as housekeeping gene and mRNA expression ratio was normalized to control (wt) mean values set to 1. Error bars indicate SE. *: P<. 05; **: P<. 01; ***: P<. 001.
(TIFF)

S5 Fig Tfr1 and FtL protein levels in cells isolated from *Hjv* KO mice. HCs (**A**), KCs (**B**) and LSECs (**C**) were isolated from wild type (wt) and *Hjv* KO mice. Cells were lysed in Lysis Buffer as described in Material and Methods and protein extracts were loaded onto a 12% SDS PAGE and processed for Western Blot analysis. Anti-Tfr1 and anti-FtL Ab were used to detect endogenous Tfr1 and FtL respectively. Equal protein transfer were verified by Ponceau staining.
(TIFF)

S6 Fig Iron parameters and hepcidin levels in *Tmprss6* KO mice. Non-heme liver (LIC, **A**) and spleen (SIC, **D**) iron content and transferrin saturation (TS, **B**) in wild type (wt) and *Tmprss6* KO mice (4–6 mice/group). Hepcidin mRNA expression (*Hamp*, **C**) was evaluated in isolated HCs by qRT-PCR relative to the housekeeping *Hprt1* gene. mRNA expression was normalized to control (wt) mean values set to 1. Error bars indicate SE. *: P<. 05; **: P<. 01; ns: not significant.
(TIFF)

S7 Fig Hepatocytes *Tmprss6* expression in iron-loading and iron deficient conditions. *Tmprss6* expression was measured by qRT-PCR in HCs isolated from: **A**) mice maintained 3 weeks an iron balanced (IB), iron deficient (ID) and iron loading (IL) diet (6 mice/ group). mRNA expression ratio was normalized to a control (IB) mean values set to 1. **B**) mice maintained 2 weeks an ID diet and then treated with 1 day ID (ID-1 day), IB (IB-1 day), IL (IL-1 day) diet (4 mice/group). **C**) wild type (wt) and *Hjv* KO mice (4 mice/group). *Hprt1* was used as housekeeping gene for mRNA quantification. Error bars indicate SE. ns: not significant.
(TIFF)

S8 Fig *Bmp6* and *Tfr1* expression. *Bmp6* (**A**, **C**, **E**) and *Tfr1* (**B**, **D**, **F**) expression were assessed by qRT-PCR in liver cells population from C57BL/6, maintained a 3 weeks iron balanced (IB), iron deficient (ID) and iron loading (IL) diets (**A**, **B**), *Hjv* KO mice (**C**, **D**) and *Tmprss6* KO animals (**E**, **F**) male mice. *Hprt1* was used as housekeeping gene for mRNA quantification. Error bars indicate SE.
(TIFF)

S1 Table Oligonucleotides used for qRT-PCR. (DOCX)
