# Supplementary figures and images for: Bmp6 Expression in Murine Liver Non Parenchymal Cells: A Mechanism to Control their High Iron Exporter Activity and Protect Hepatocytes from Iron Overload?
Source: PLoS One. 2015 Apr 10;10(4):e0122696. doi: 10.1371/journal.pone.0122696 (PMC4393274; doi:10.1371/journal.pone.0122696)

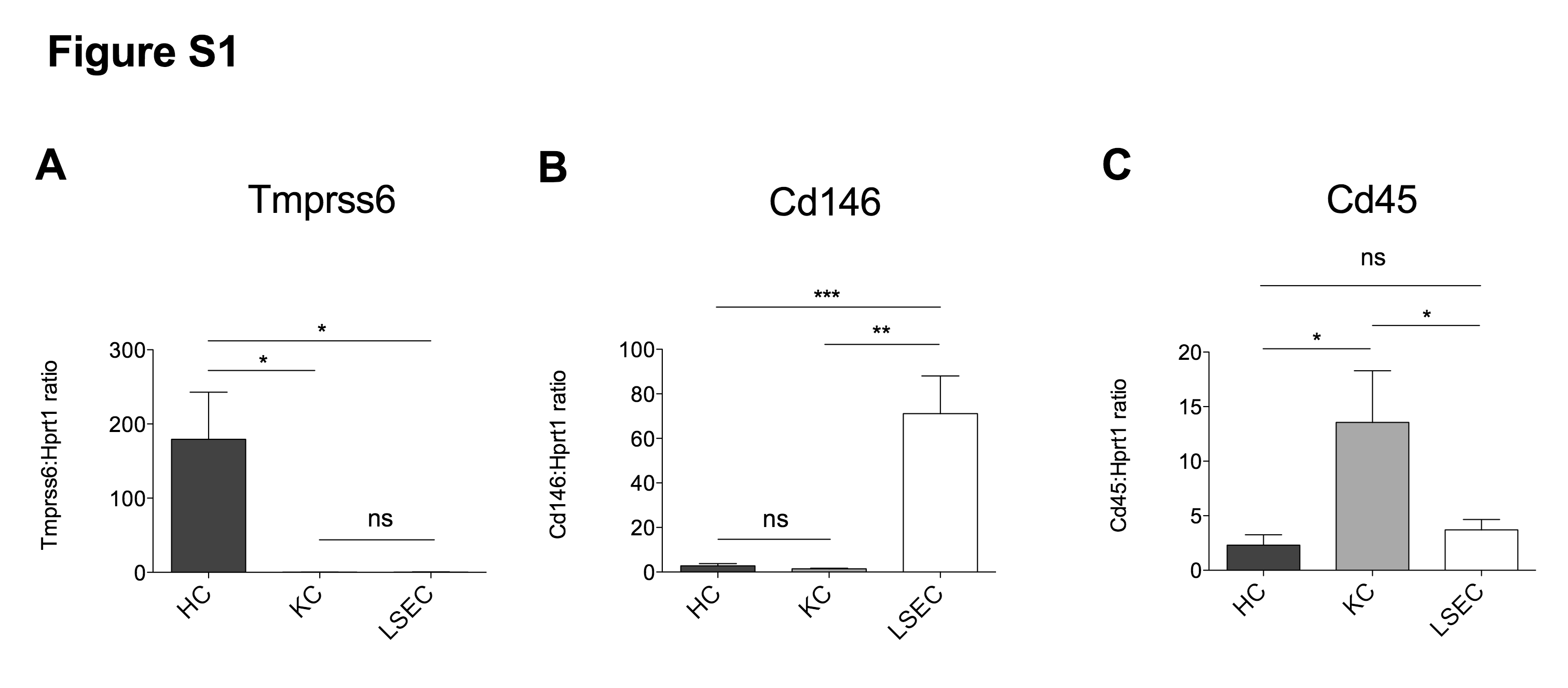

Supplement: S1 Fig — Liver cells were isolated from 4 mice. Tmprss6 (A), Cd146 (B) and Cd45 (C) mRNA expression was quantified by qRT-PCR relative to housekeeping Hprt1 mRNA to evaluate the purity of HCs, KCs and LSECs, respectively. Error bars indicate SE. *: P<. 05; **: P<. 01; ***: P<. 001. (TIFF) [file pone.0122696.s001.tiff]

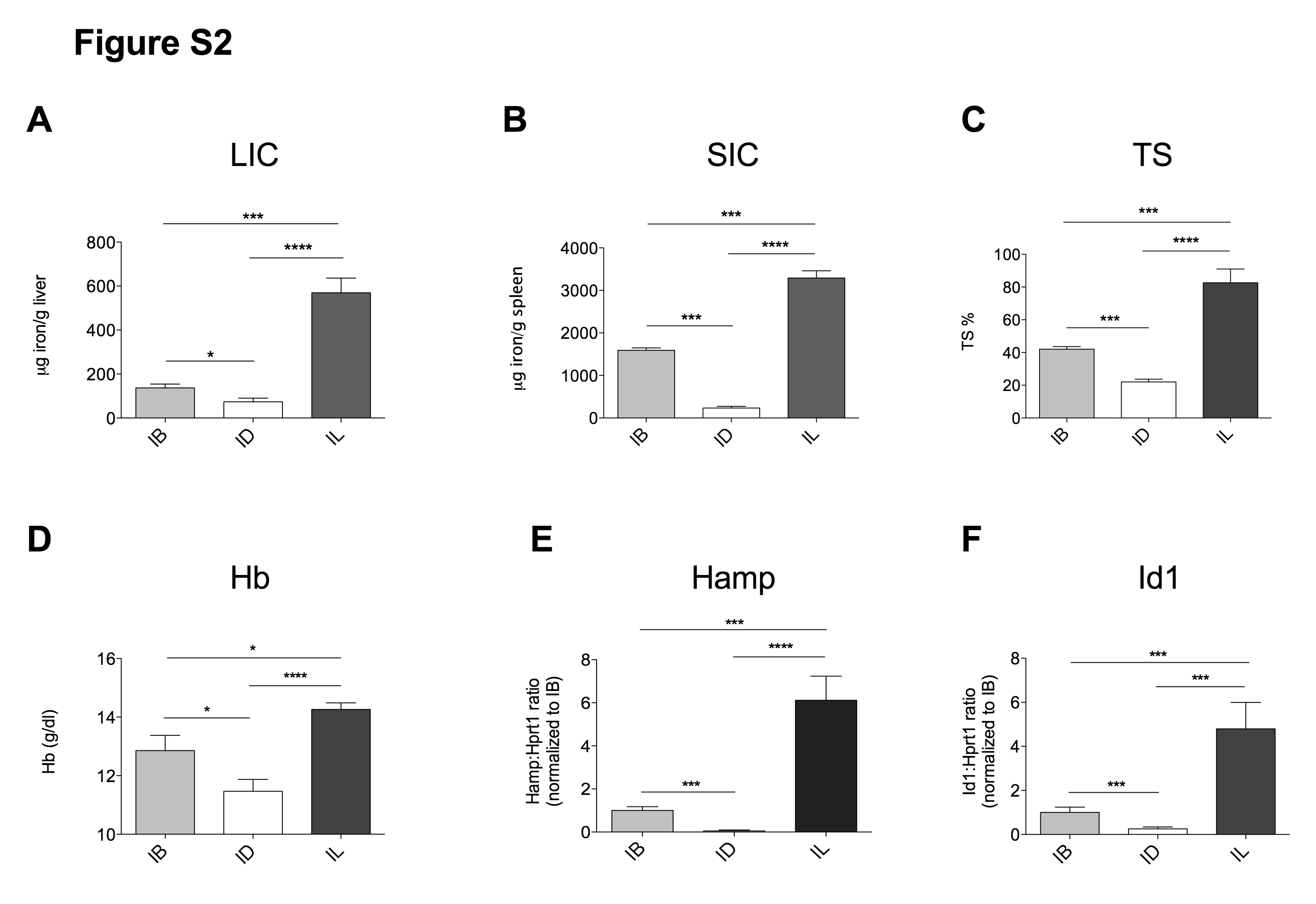

Supplement: S2 Fig — Mice were fed an iron balanced (IB), iron deficient (ID) and iron loading (IL) diet for 3 weeks (6–8 mice/group). Non-heme liver (LIC, A), spleen (SIC, B) iron content, transferrin saturation (TS, C) and hemoglobin levels (Hb, D) are shown. In isolated hepatocytes hepcidin (Hamp) and Id1 mRNA expression was quantified by qRT-PCR relative to housekeeping Hprt1 gene. mRNA expression ratio was normalized setting control (IB) mean value to 1. Error bars indicate SE. *: P<. 05; **: P<. 01; ***: P<. 001. (TIFF) [file pone.0122696.s002.tiff]

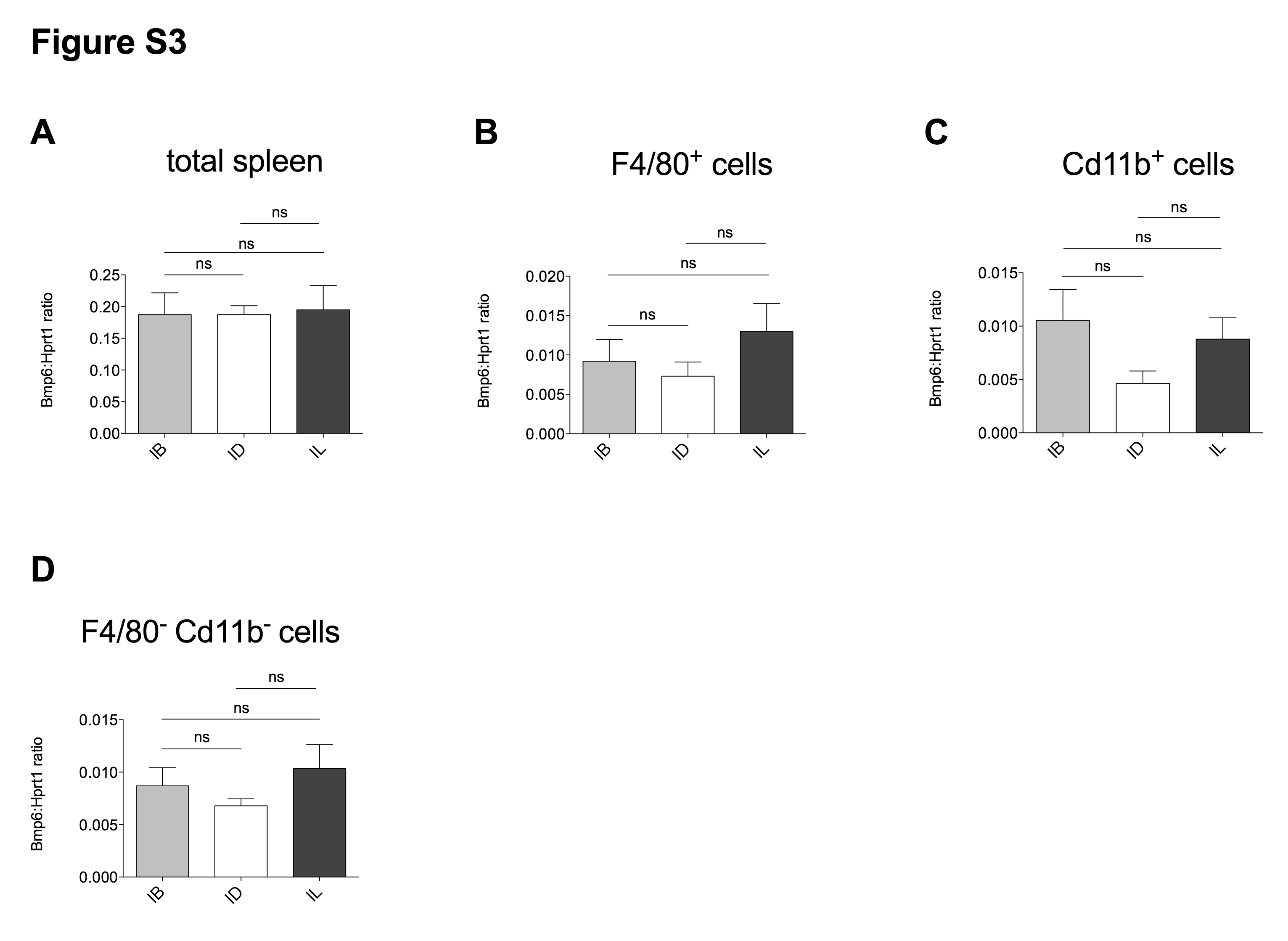

Supplement: S3 Fig — Spleen and spleen-derived cells were isolated from mice maintained an iron balanced (IB), iron deficient (ID) and iron loading (IL) diet for 3 weeks. Bmp6 expression from total spleen (A, 3–4 mice), from F4/80+ cells (B, 6 mice), from Cd11b+ cells (C, 6 mice) and from negative fractions (D, 6 mice) was quantified by qRT-PCR relative to Hprt1 as the housekeeping gene. Error bars indicate SE. ns: not significant. (TIFF) [file pone.0122696.s003.tiff]

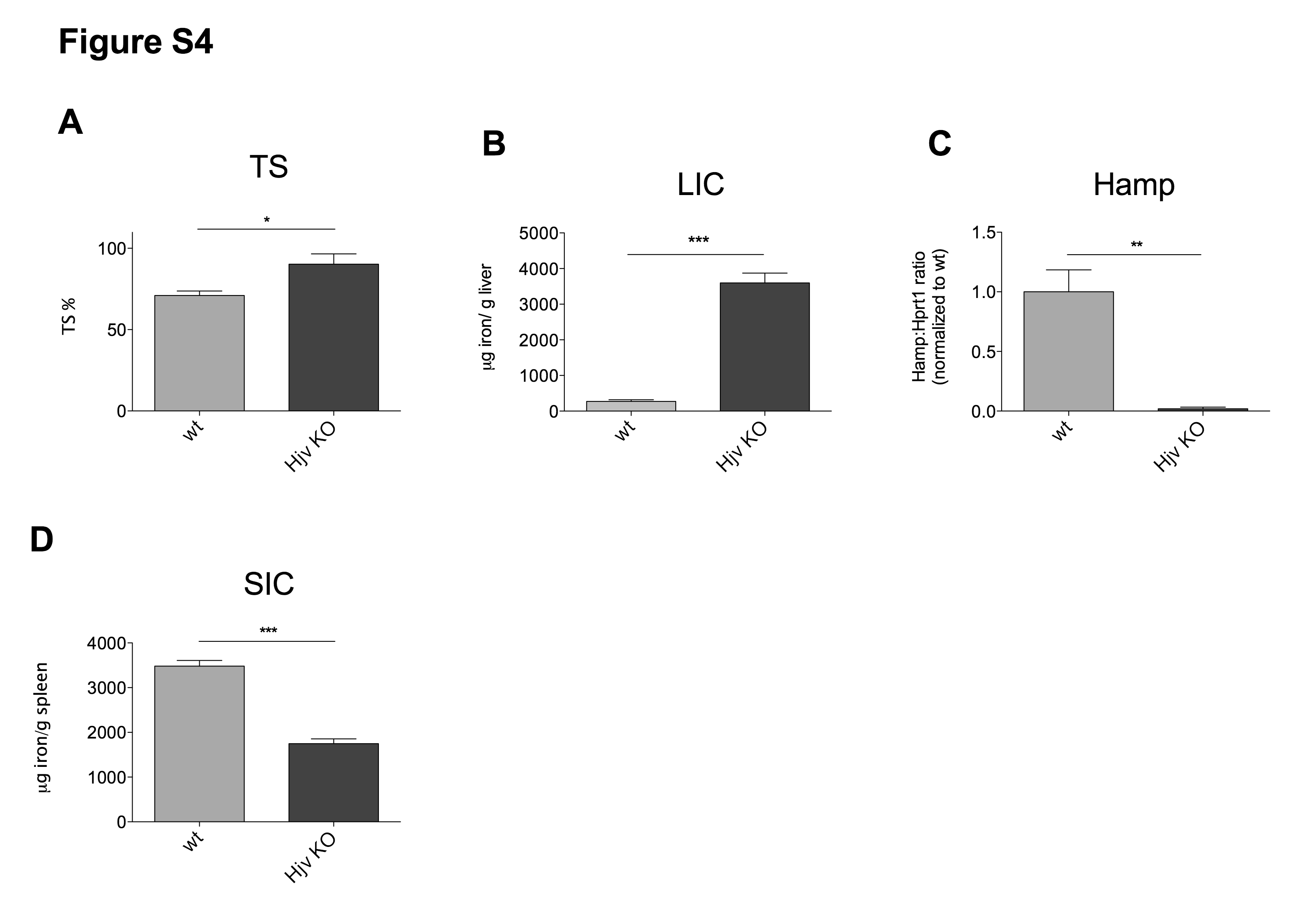

Supplement: S4 Fig — Transferrin saturation (TS, A), non-heme sliver iron content (LIC, B) and non-heme spleen iron content (SIC, D) were measured in wild type (wt) and Hjv KO animals (4 mice/group). In isolated HCs, hepcidin (Hamp, C) expression was measured by qRT-PCR, using Hprt1 as housekeeping gene and mRNA expression ratio was normalized to control (wt) mean values set to 1. Error bars indicate SE. *: P<. 05; **: P<. 01; ***: P<. 001. (TIFF) [file pone.0122696.s004.tiff]

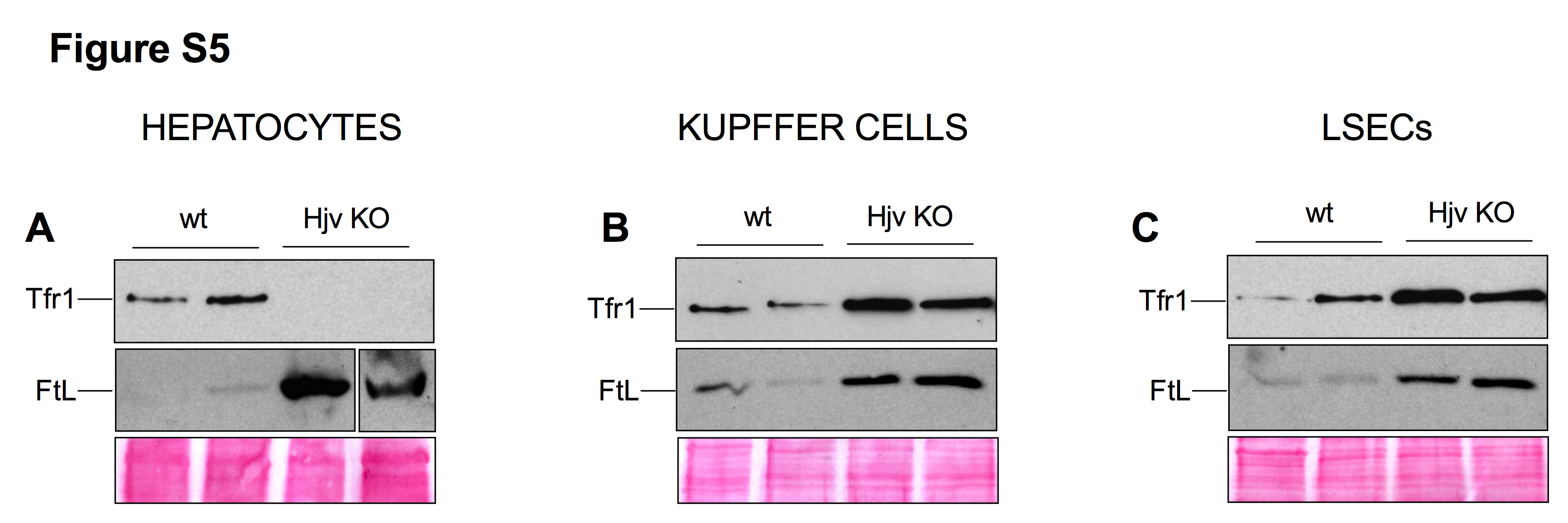

Supplement: S5 Fig — HCs (A), KCs (B) and LSECs (C) were isolated from wild type (wt) and Hjv KO mice. Cells were lysed in Lysis Buffer as described in Material and Methods and protein extracts were loaded onto a 12% SDS PAGE and processed for Western Blot analysis. Anti-Tfr1 and anti-FtL Ab were used to detect endogenous Tfr1 and FtL respectively. Equal protein transfer were verified by Ponceau staining. (TIFF) [file pone.0122696.s005.tiff]

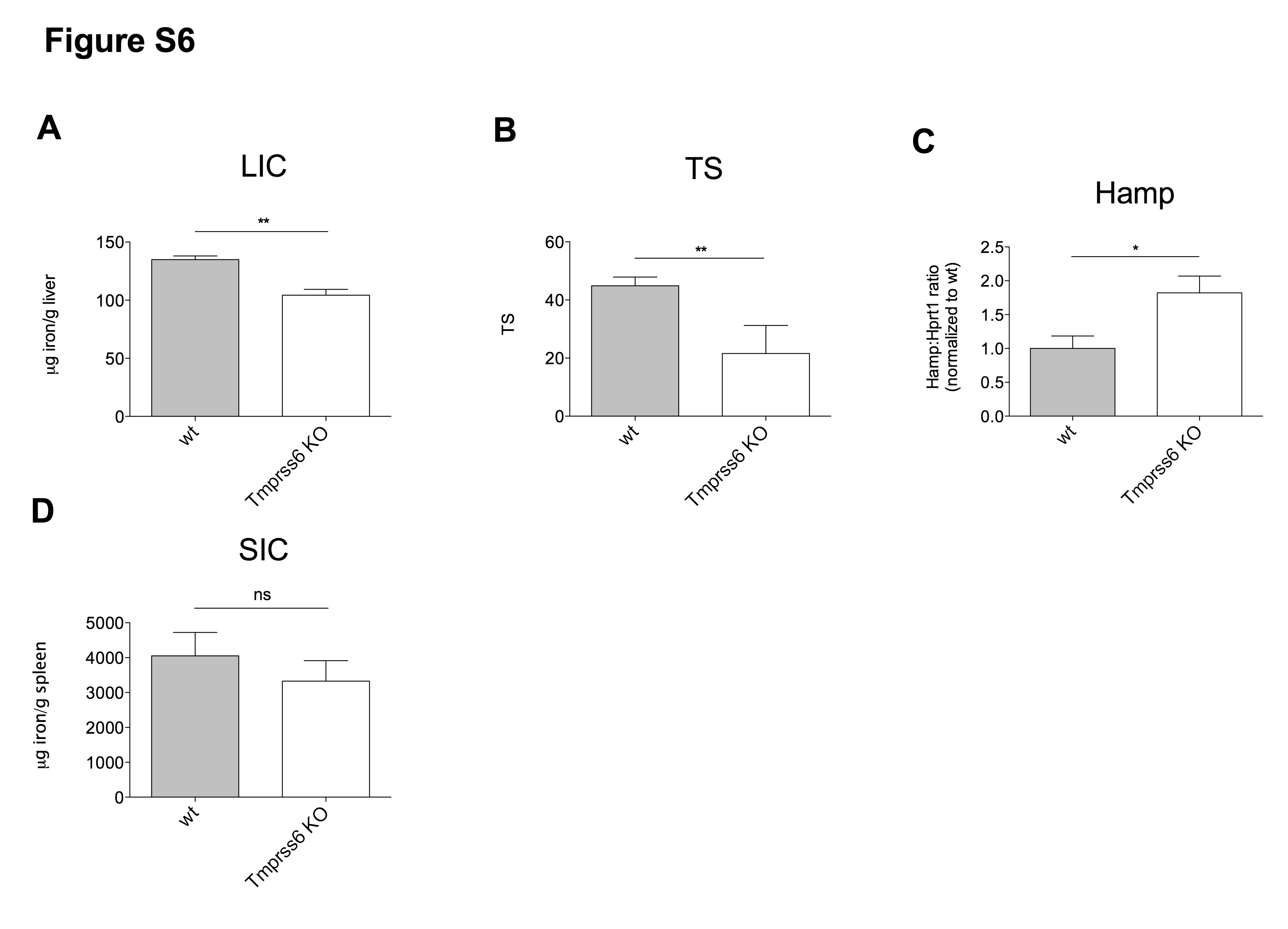

Supplement: S6 Fig — Non-heme liver (LIC, A) and spleen (SIC, D) iron content and transferrin saturation (TS, B) in wild type (wt) and Tmprss6 KO mice (4–6 mice/group). Hepcidin mRNA expression (Hamp, C) was evaluated in isolated HCs by qRT-PCR relative to the housekeeping Hprt1 gene. mRNA expression was normalized to control (wt) mean values set to 1. Error bars indicate SE. *: P<. 05; **: P<. 01; ns: not significant. (TIFF) [file pone.0122696.s006.tiff]

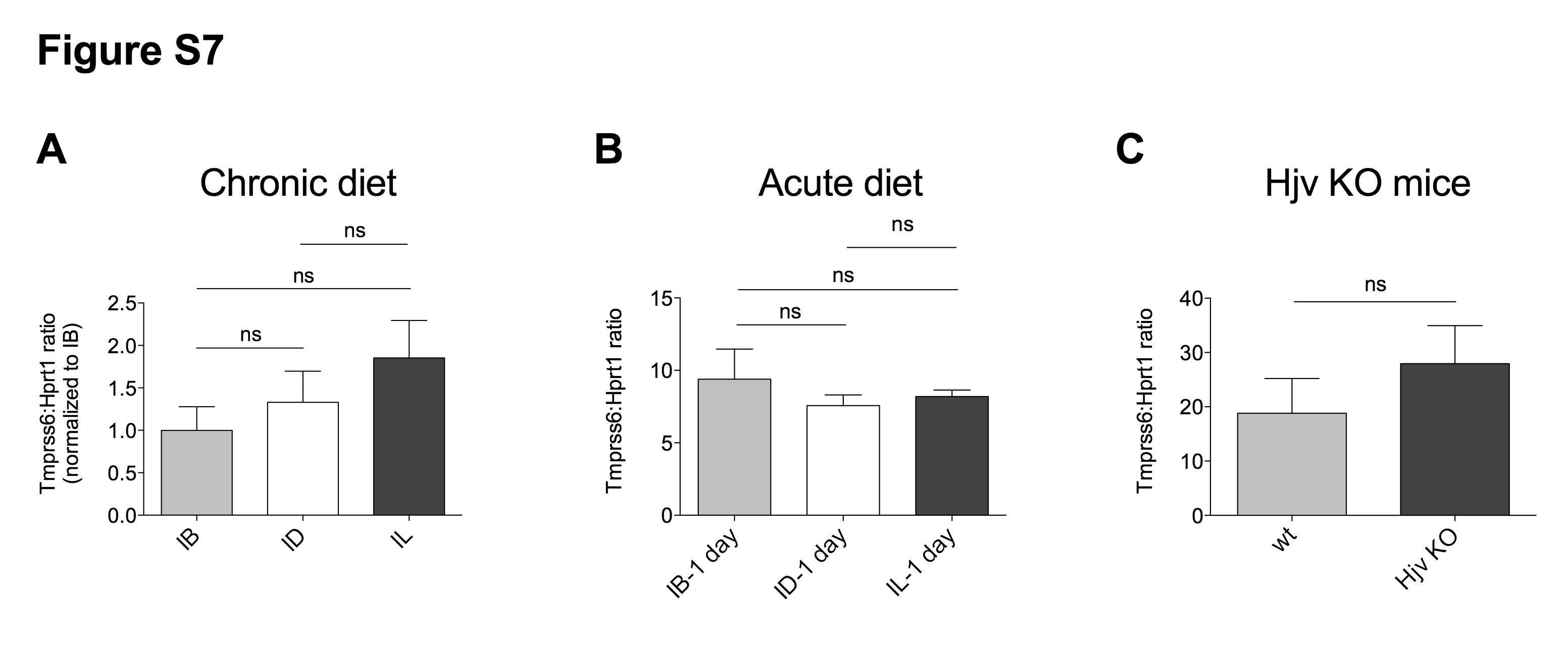

Supplement: S7 Fig — Tmprss6 expression was measured by qRT-PCR in HCs isolated from: A) mice maintained 3 weeks an iron balanced (IB), iron deficient (ID) and iron loading (IL) diet (6 mice/ group). mRNA expression ratio was normalized to a control (IB) mean values set to 1. B) mice maintained 2 weeks an ID diet and then treated with 1 day ID (ID-1 day), IB (IB-1 day), IL (IL-1 day) diet (4 mice/group). C) wild type (wt) and Hjv KO mice (4 mice/group). Hprt1 was used as housekeeping gene for mRNA quantification. Error bars indicate SE. ns: not significant. (TIFF) [file pone.0122696.s007.tiff]

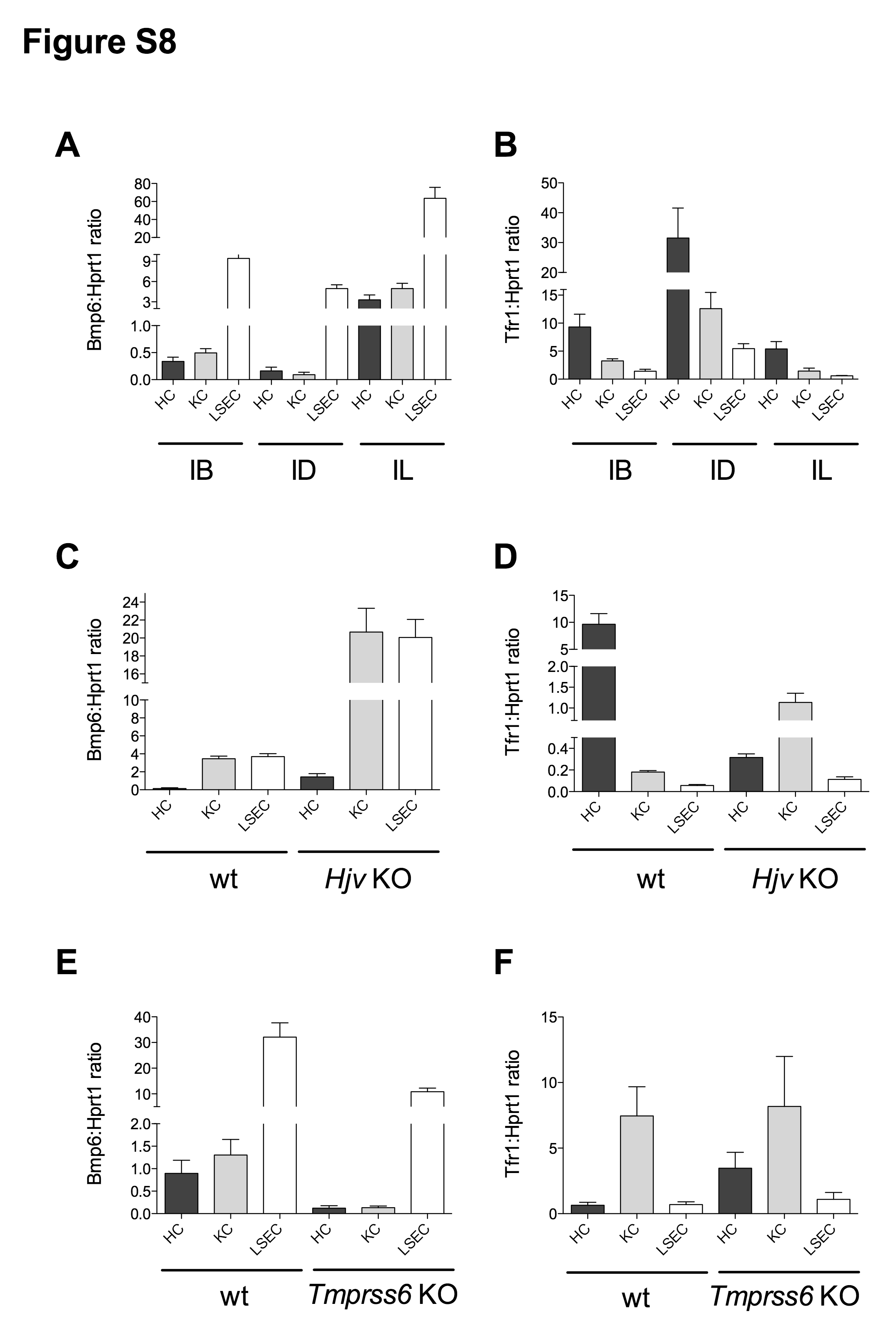

Supplement: S8 Fig — Bmp6 (A, C, E) and Tfr1 (B, D, F) expression were assessed by qRT-PCR in liver cells population from C57BL/6, maintained a 3 weeks iron balanced (IB), iron deficient (ID) and iron loading (IL) diets (A, B), Hjv KO mice (C, D) and Tmprss6 KO animals (E, F) male mice. Hprt1 was used as housekeeping gene for mRNA quantification. Error bars indicate SE. (TIFF) [file pone.0122696.s008.tiff]
