# Supplementary material for: Bmp6 Expression in Murine Liver Non Parenchymal Cells: A Mechanism to Control their High Iron Exporter Activity and Protect Hepatocytes from Iron Overload?
Source: PLoS One. 2015 Apr 10;10(4):e0122696. doi: 10.1371/journal.pone.0122696 (PMC4393274; doi:10.1371/journal.pone.0122696)
Supplement: S1 Table — (DOCX) [file pone.0122696.s009.docx]

**S1 Table. Oligonucleotides used for qRT-PCR.**

| **Name** | **Id** |
| --- | --- |
| *Bmp6* | Mm01332882_m1 |
| *Cd45* | Mm01293575_m1 |
| *Cd146* | Mm00522397-m1 |
| *Id1* | Mm00775963_g1 |
| *Hamp* | Mm00519025_m1 |
| *Hprt1* | Mm01318743_m1 |
| *Slc40a1* | Mm00489837_m1 |
| *Tfr1* | Mm00441941_m1 |
| *Tmprss6* | Mm0551119_m1 |
